# Estimation of Shape Error with Monitoring Signals

**DOI:** 10.3390/s23239416

**Published:** 2023-11-26

**Authors:** Hyein Kim, Soohyun Nam, Eunseok Nam

**Affiliations:** 1 Smart Manufacturing System R&D Department, Korea Institute of Industrial Technology (KITECH), Cheonan 31056, Republic of Korea; hyeinkim@kitech.re.kr; 2 Digital Transformation R&D Department, Korea Institute of Industrial Technology (KITECH), Ansan 15588, Republic of Korea; esnam86@kitech.re.kr

**Keywords:** shape error, error compensation system, on-machining measurement system, multi-layer perceptron, sensor fusion

## Abstract

Recently, extensive research has actively been conducted in relation to intelligent manufacturing systems. During the machining process, various factors, such as geometric errors, vibrations, and cutting force fluctuations, lead to shape errors. When a shape error exceeds the tolerance, it results in improper assembly or functionality issues in the assembled part. Predicting shape errors before or during the machining process helps increase production efficiency. In this paper, we propose a methodology that uses monitoring signals and on-machine measurement (OMM) results to predict machining quality in real time. We investigate the correlation between monitoring signals and OMM results and then construct a machine learning model for shape error estimation. The developed model implements a tool offset compensation strategy. The performance of the proposed method is evaluated under various sliding window sizes and the compensation weights. The experimental results confirmed that the proposed algorithm is effective for obtaining a uniform machining quality.

## 1. Introduction

In parallel with advancements in production technology, there has been a notable rise in the demand for high-precision machined products. It is known that shape error can be attributed to various factors, such as tracking errors in the control system, thermal errors, and errors caused by cutting force fluctuation [1,2]. Extensive research has been dedicated to identifying error sources and achieving error compensation [1,2,3,4,5,6,7,8]. Schmitz et al. measured errors resulting from various sources in high-speed milling and assessed the influence of each error source comparatively [5]. Kong et al. developed a kinematics model of a two-axis ultra-precision machining system that enabled the calculation and compensation of shape error and generated a novel tool path [6]. However, the utilization of modeling to predict shape errors requires a substantial database of experiments. Furthermore, error compensation deteriorates when the accuracy of various models, including machining errors and thermal errors, is not guaranteed. To address these limitations, an offline error compensation method through an inspection process has been proposed [7,8]. Lo et al. measured an error using a coordinate measuring machine (CMM) after the machining process was finished, and these errors were then compensated in subsequent processes [7]. Raksiri et al. utilized a laser interferometer to measure geometric errors and estimated the machining errors caused by cutting forces for error compensation [8]. However, those methods have drawbacks, such as spatial constraints and decreased production efficiency due to repetitive measurement processes. Recently, the on-machine measurement (OMM) system has gained considerable attention as an innovative inspection technique with the advantages of high efficiency and high stability [9,10]. With the OMM system, the machining process and the inspection process can be conducted with the same machine tool. Cho et al. implemented an agile machining error compensation method for flat-end milling processes based on a polynomial neural network trained by OMM inspection data [9]. Ge et al. developed an integrated method that combines an OMM system, error modeling, and a real-time error compensation process [10]. Those methods are effective for simple-shape products, but their application becomes challenging for intricate-shape products in which cutting conditions randomly change. However, existing studies utilizing OMM data for shape error estimation based on various methods also have limitations. In a recent smart factory, variable machines and equipment were interconnected and communicated with each other to enable real-time monitoring, analysis, and decision-making [11]. Therefore, a time latency problem naturally occurs. In other words, real-time responsiveness is challenging. Applying real-time shape error estimation and compensation using computational modeling and complex algorithms has not been achieved. Furthermore, adaptability and generalization to various cutting conditions have been limited. A new learning model should be constructed in which cutting conditions are changed or the cutting type is converted. These iterative processes require a significant, extensive computational cost as well as frequent system maintenance. To address these limitations, this study aims to develop a real-time shape error estimation and compensation methodology. To secure a real-time response, we utilize the monitoring signals and simple analysis method. For detail, the proposed methodologies utilize a sliding window and MLP (multi-layer perceptron) model using monitoring signals and OMM measurement data. Firstly, the complex relationship between monitoring signals and OMM measurement data is investigated. Secondly, we estimate the shape error and then calculate the error compensation value. Cutting experiments are carried out to evaluate the performance of the proposed methodologies.

## 2. Shape Error Compensation Methodology

This section describes our shape error compensation methodology and data acquisition system in detail. In Section 2.1, an outline of the proposed methodology is presented. In Section 2.2 and Section 2.3, the monitoring system and the OMM system utilized for this study are described. In Section 2.4, the experimental setup and conditions are introduced.

### 2.1. Proposed Error Compensation Methodology

**Figure 1:**
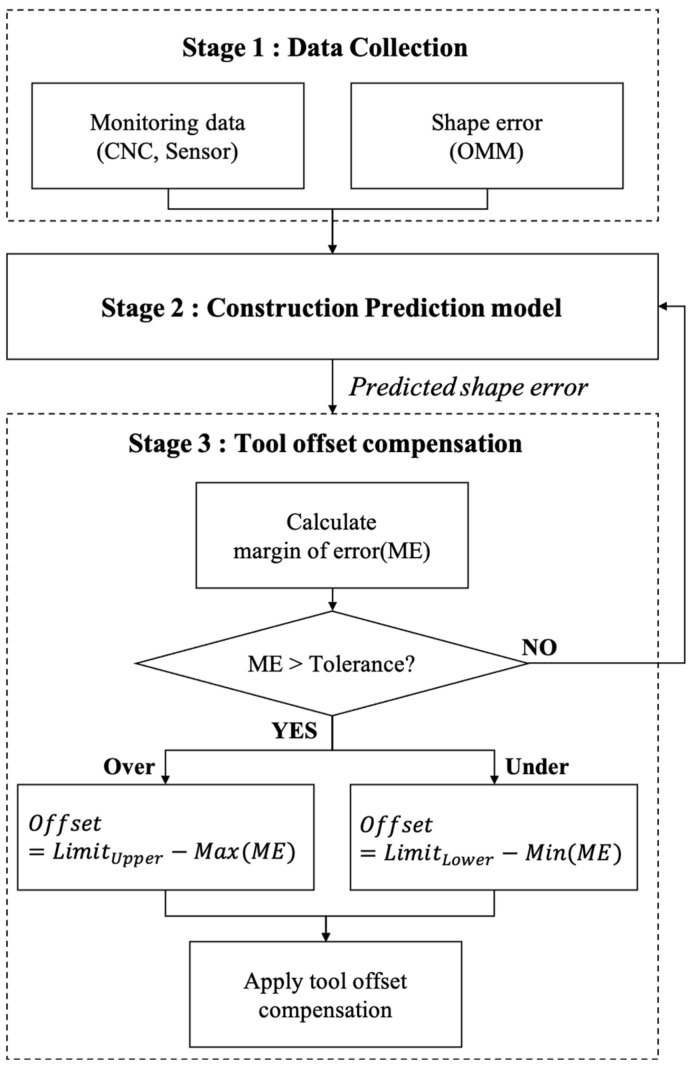
Schematic diagram of proposed method.

Figure 1 depicts the schematic diagram of the proposed error compensation methodology. The data utilized in this method are categorized into two types: firstly, monitoring data gathered during the machining process, and secondly, shape error data measured by the OMM system once each machining process has been completed. In the initial step, a signal selection process is carried out to identify which monitoring signals (from both CNC and sensors) are suitable as input features. This process is conducted by correlation analysis between these signals and the measured shape error. Subsequently, a prediction model is established for estimating the shape error. We define the estimated shape error as the ‘margin of error (ME)’. If the ME exceeds the specified tolerance, we initiate the tool offset compensation process. We implement different tool compensation methods based on whether ME surpasses the upper or lower tolerance limit.

Error compensation utilizes the tool diameter compensation function provided by a FANUC controller (FANUC Corp., Series 31i-MODEL B, Yamanashi, Japan). This function adjusts the tool path in the radial direction of the tool by referring to the compensation value assigned to the macro variable within the controller. This function is user-friendly, as it involves updating the compensation value without the necessity of generating a new NC program for error compensation. Previous studies have already confirmed the effectiveness of error reduction through tool path compensation [7,9,10,11,12].

### 2.2. Monitoring System

**Figure 2:**
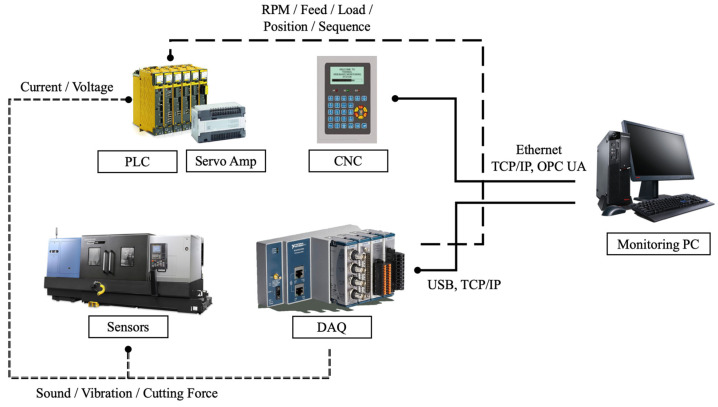
Schematic diagram of monitoring system.

Figure 2 illustrates a schematic diagram of the monitoring system, and Table 1 provides a list of monitoring data obtained from both CNC and various sensors. CNC data are sampled at a frequency of 20 Hz through the FANUC controller, whereas sensor data are sampled at a significantly higher frequency of 12,800 Hz via a data acquisition system (DAQ). Motor drive current and applied voltage data are obtained by attaching a hall sensor (Seri2B., SCV-U2, Gyeongbuk, Republic of Korea) and a transducer (Seri2B., IVS-D4, Gyeongbuk, Republic of Korea) to the cables of the spindle motor and the feed drive motor on the machine tool distribution board. An accelerometer (DYTRAN Instrument Inc., 3211B1-30, Los Angeles, CA, USA) is installed on the spindle housing to collect *X*- and *Y*-axis vibration data that appear during the machining process. Additionally, a microphone (PCB Electronics Inc., 130A24, Hong Kong, China) is attached to the outer wall of the machine tool to capture sound data.

### 2.3. OMM System

**Figure 3:**
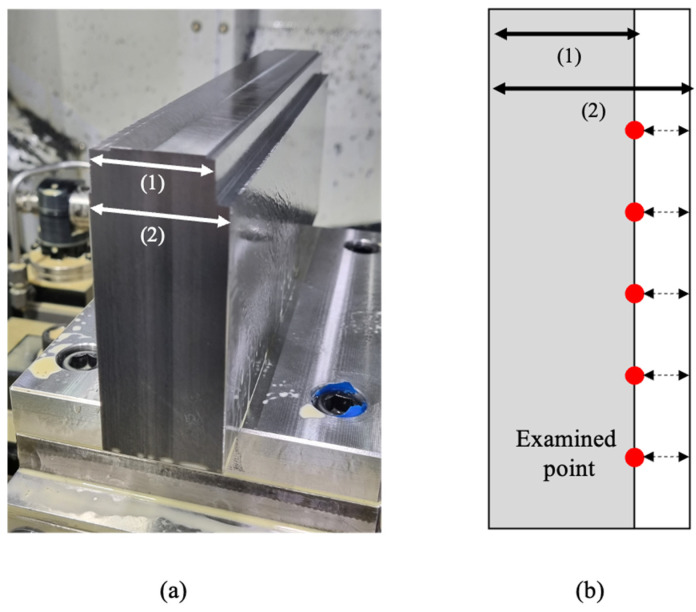
OMM measurement. (**a**) Target workpiece. (**b**) Examined point of workpiece.

On-machine measurement (OMM) refers to measurement and inspection that takes place directly on a machine tool after the machine has finished its task (Figure 3). After each machining process, the shape of the product is examined using a touch probe (Renishaw Inc., OMP400, Hong Kong, China). The system detects the force resulting from the contact between the ball at the end of the stylus and the workpiece. It records the mechanical coordinates of all contact points and determines the shape by computing the distance between these contact points. Note that this computed distance involves the stylus ball radius. To ensure accuracy, we calibrate the stylus ball radius utilizing a reference specimen. Moreover, when attaching the touch probe onto the tool shank or holder, there is potential for runout due to the misalignment between the center of the stylus and the center of the spindle. We align these central axes meticulously to mitigate inaccuracies.

The measurement of shape errors was carried out by performing residual width measurements after the slot milling for 20 iterations. Measurements were conducted five times per single machining operation, resulting in a total of 100 measurement data points that were utilized in the study.

### 2.4. Experimental Setup and Conditions

A sequence of machining experiments was conducted utilizing a 3-axis machine tool (DN Solutions Co., Ltd., NX-5500II, Changwon, Republic of Korea) to validate the proposed methodology. The experimental conditions are detailed in Table 2. The specimen of C45E4 was machined in a straight manner. Subsequent to the machining process, the remaining part was measured using a touch probe to obtain the shape dimensions. We calculated the shape error by comparing these measurement results with the target shape information. Throughout one straight-line machining operation, measurements were taken at five equidistant intervals, and this procedure was iterated 20 times. Prior to each iteration of the experiment, the upper surface was face-milled, and the side surface was shoulder-milled two times to rectify any potential tilt that could have occurred during workpiece installation. From the total machining processes, we confirmed a shape error ranging from a minimum of 40 μm to a maximum of 119 μm.

## 3. Data Processing and Feature Extraction

This section describes the data processing method and feature extraction process in detail. In Section 3.1, the signal preprocessing of collected data is presented. In Section 3.2 describe the feature selection methods utilized for this study.

### 3.1. Signal Preprocessing

**Figure 4:**
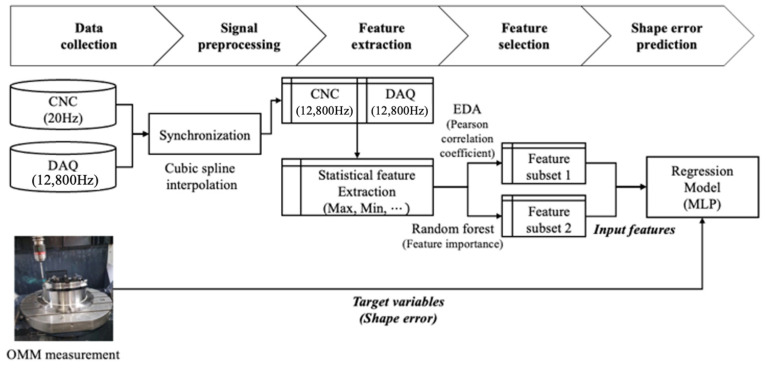
Overall data processing.

Figure 4 illustrates the schematic diagram of overall data processing. In order to ensure the compatibility of data collected from both the FANUC controller and the DAQ board, which operate at different sampling rates, a synchronization process was carried out to align them. To accomplish this, CNC data were interpolated at a frequency of 12,800 Hz through cubic spline interpolation. We integrated them with the DAQ data to create a unified dataset. Seven descriptive statistics, namely root mean square (RMS), maximum (Max), minimum (Min), mean, kurtosis, skewness, and standard deviation, were extracted from the interpolated data at 0.1 s intervals. This procedure entailed converting raw data gathered from various sensors into a format that is suitable for application as input features in a machine learning model. This offers the advantage of reducing interdependence among features by decreasing the dimensionality of raw data while simultaneously preserving the inherent characteristics of time series data [13]. 

### 3.2. Feature Selection

Utilizing an extensive amount of the extracted data as-is could increase the risk of overfitting and potentially lead to a deteriorate the machine learning model performance [14]. In order to only select features having a high correlation with shape error among the extracted dataset, we performed a feature selection process. The feature selection methods are broadly divided into three types. The first is the Filter method, which employs statistical evaluation techniques. The second is the Embedded method, which selects features significantly influencing the model accuracy. The last is the Wrapper method, which creates subsets with the highest performance by generating all possible combinations of a given feature set [15]. Among these methods, in this study, we conducted the feature selection process using the Filter and Embedded methods.

#### 3.2.1. Filter Method

**Figure 5:**
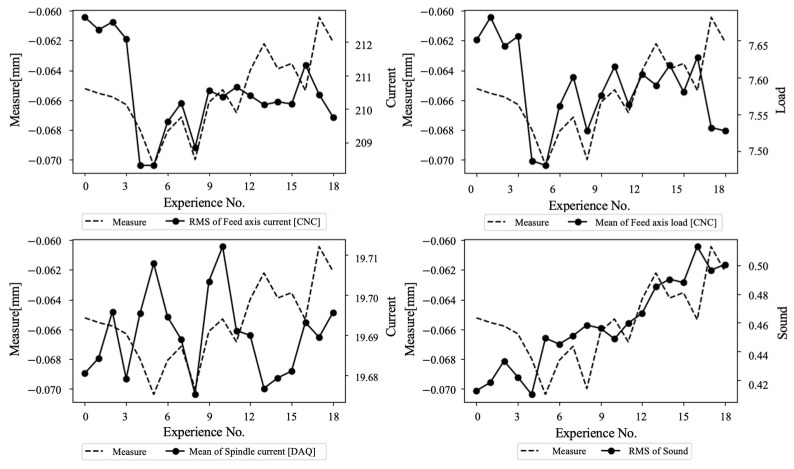
Results of Pearson correlation coefficient analysis.

The feature selection process utilizing the Pearson correlation coefficient is performed as expressed by Equation (1). The Pearson correlation coefficient is a concept used when assessing similarity between input vectors. The closer the r value is to 1, the higher the correlation, and the larger this value, the higher the importance of the feature. As a result, as shown in Figure 5, it is confirmed that the mean values of feed axis current, feed axis load, spindle axis current, and RMS of sound denote the highest correlation with shape error. They are selected as input features.

(1) $$r=\frac{\sum (x-\bar{x})(y-\bar{y})}{\sqrt{{\sum (x-\bar{x})}^{2}{\sum (y-\bar{y})}^{2}}}$$

#### 3.2.2. Embedded Method

**Figure 6:**
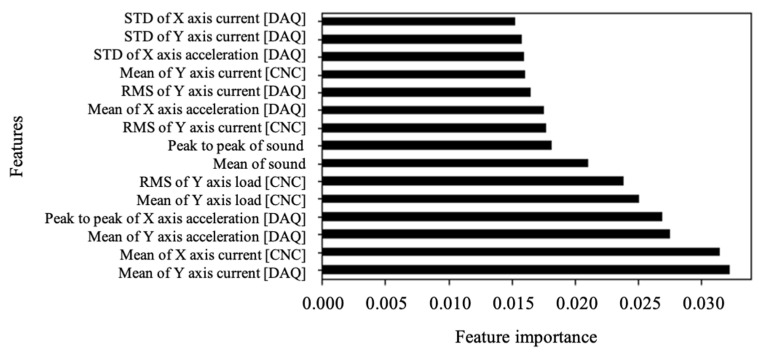
Feature importance plot.

Additional features are selected through the Embedded method, which assesses the importance and influence of the feature considering the characteristics of the model [16]. Random forest (RF) is an ensemble method that generates multiple decision trees and synthesizes the results. It offers the advantage of providing feature importance and assuring superior predictive performance, particularly in scenarios with many explanatory variables. Feature importance is a value that indicates the influence of a specific independent value on the prediction results. The larger the value, the greater the contribution to prediction accuracy. Figure 6 shows the RF feature importance plot. Among these, the top 10 features do not overlap with the results obtained through the Filter method.

## 4. Shape Error Compensation Results

This section describes the construction of the machine learning model and the shape error compensation results in detail. In Section 4.1, the constructed MLP model is presented. In Section 4.2, the shape error compensation results are discussed.

### 4.1. MLP Model Construction

**Figure 7:**
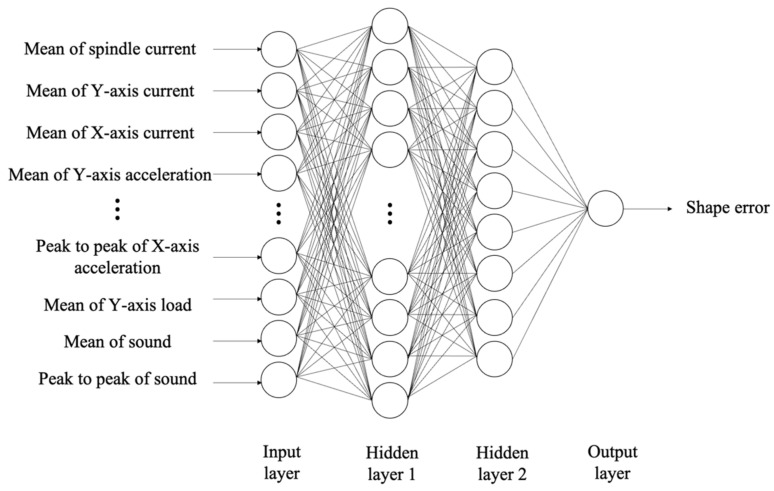
MLP architecture.

The MLP model, as depicted in Figure 7, is an artificial neural network with multiple hidden layers, offering a structure capable of modeling complex nonlinear relationships. Each neuron is connected to all neurons in the preceding layer, and these connections are assigned weights. The input neuron ($x_{q}$) of each layer (p) is multiplied by the weights ($w_{p,q}$) of that layer and then passes through an activation function (σ). The formula of this process is expressed by Equation (2). The output obtained through this step becomes the input for the next layer (p + 1), and this process is iterated until it reaches the output layer, resulting in the final output.

(2) $$output=\sigma \sum_{q}w_{p,q}x_{q}+bias.\;$$

We utilized the backpropagation algorithm for MLP training and used MLP Regressor in Python Keras for data analysis. The 14 variables obtained in the feature selection process were utilized for input features. The hyperparameters that users need to manually input when designing the model were set using grid search, as shown in Table 3 [17].

**Figure 8:**
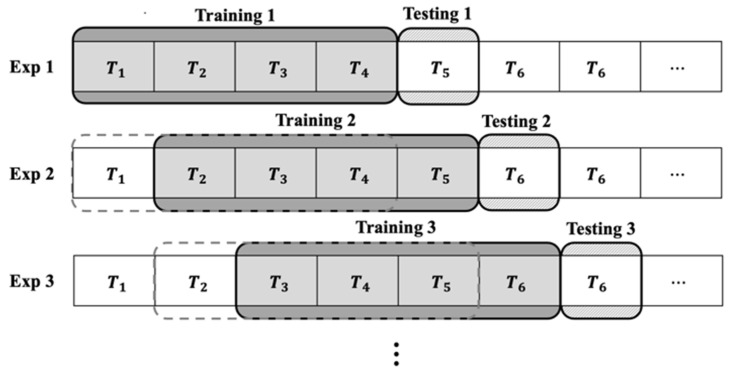
Schematic diagram of sliding window approach.

According to tool wear progress, cutting force increases due to blunt tool edges, which may lead to a discrepancy between the weights of the previously trained model and the data affected by tool wear. We utilized the sliding window approach [18] to mitigate the influence of older data and enhance the influence of recent data, as shown in Figure 8. We divided the data into 40 segments ($T_{1}$~$T_{40}$) in time series order. To assess the estimation accuracy of the proposed methodology, a training model was constructed using data from $T_{n}$ to $T_{n+3}$ and the shape error at $T_{n+4}$ was estimated. The estimation accuracy was evaluated by $R^{2}$ score expressed by Equation (3). This index indicates how well the independent variables explain the variation in the dependent variable. The closer it is to 1, the higher the model performance.

(3) $$R^{2}=1-\frac{\sum {\left(y_{i}-\hat{y_{i}}\right)}^{2}}{\sum {\left(y_{i}-\bar{y}\right)}^{2}}$$

Here, $y_{i}$ is the actual value, $\bar{y}$ is the mean of the $y_{i}$, and $\hat{y_{i}}$ is the predicted value.

**Figure 9:**
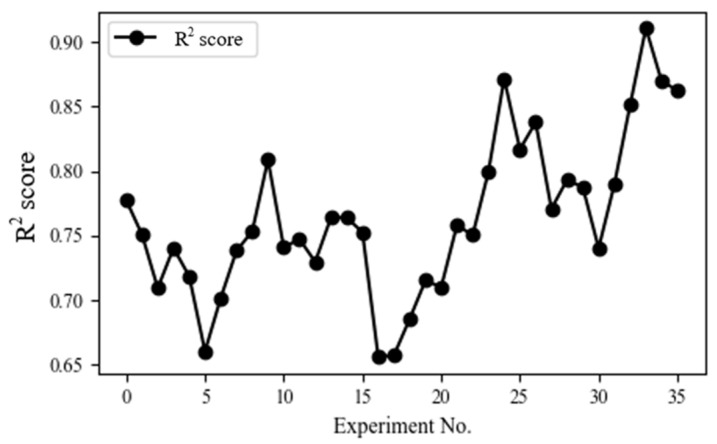
Result of $R^{2}$ score evaluation.

Figure 9 presents the $R^{2}$ score depending on the experiment number shown in Figure 8. $R^{2}$ scores ranged from 0.65 to 0.90. In the initial stages of training, the model had a relatively small $R^{2}$ score due to insufficient data. However, as time passed and the number of training iterations increased, the model was trained in complex patterns, resulting in an increase in its $R^{2}$ score.

### 4.2. Shape Error Compensation Result

**Figure 10:**
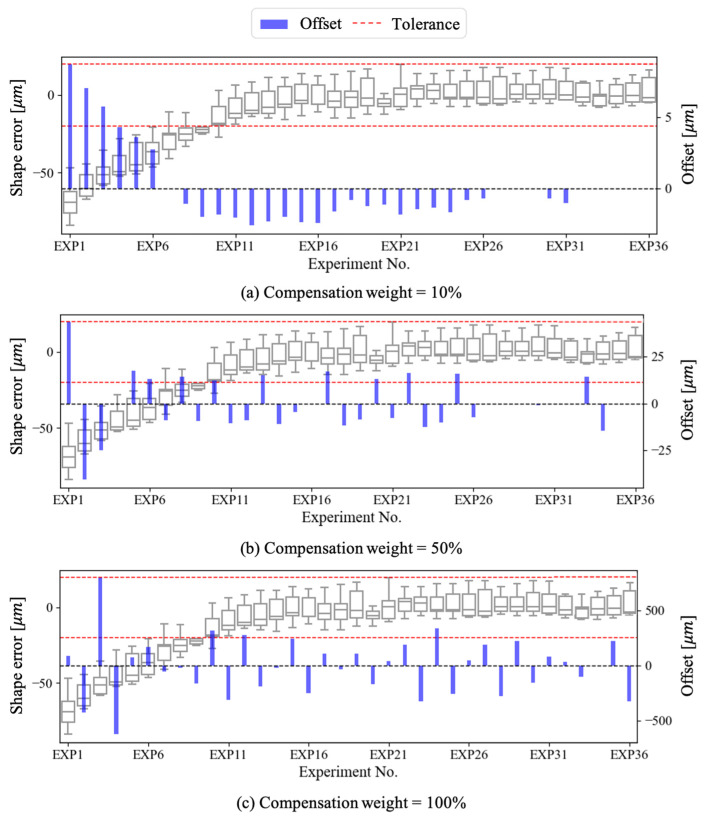
Shape error estimation and tool offset compensation.

We estimated the margin of shape error and its magnitude through the constructed model. We perform shape error compensation only when the shape error exceeded the tolerance (±20 μm). We introduced compensation weights to prevent drastic shape error compensation. We compared the results from various window sizes and compensation weights, as shown in Figure 10. When the window size was 4 and compensation weight was 50% (Figure 10a), a shape error of −70 μm occurred in Exp1 and −30 μm in Exp2. Since these values significantly exceed the tolerance, we performed tool offset compensation in a positive direction in both cases. This process was iterated, and finally, machining could be carried out within the tolerance. When the compensation weight increased by 70%, we investigated the issue, as shown in Figure 10b. From the result, we observed that excessive shape error compensation led to frequent violations of the tolerance. Lastly, we investigate the influence of window size by decreasing the window size to 1, as shown in Figure 10c. We confirm that a decreased window length reduces the data utilized for training the model, leading to a decrease in prediction performance. As a result, the margin of shape error is estimated to be wider, and frequent shape error compensation is observed. Among the results from the three conditions, the best performance was achieved with a window size of 4 and a compensation weight of 50%.

**Figure 11:**
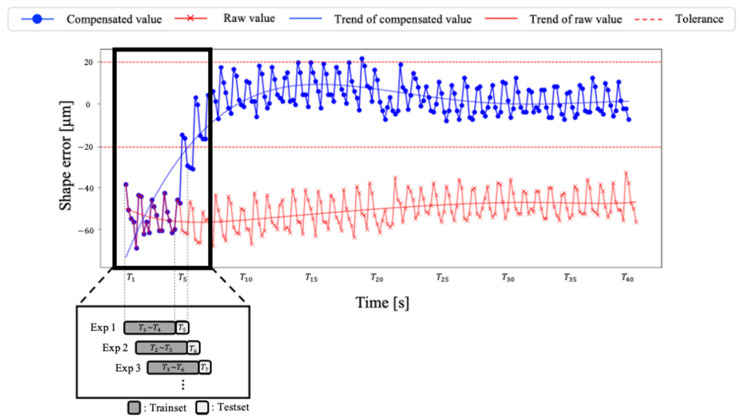
Comparison of raw values and compensated values.

Figure 11 shows a graph comparing the results before and after compensation under the conditions showing the best performance. Red lines represent raw values without compensation and blue lines represent the compensated values, respectively. In the initial section (Exp1), the initial data ($T_{1}$~$T_{4}$) are used as the training data, and hence, the results of raw values and compensation values are identical. We confirm that error estimation proceeds from $T_{5}$. From this point on, it is observed that errors occurring during machining do not exceed the tolerance, except for the initial section. This denotes that the proposed methodology effectively compensates for shape errors. Real-time and accurate shape error compensation can be only achieved using monitoring data. These findings are expected to be valuable in the manufacturing field in terms of enhancing shape precision and production efficiency.

## 5. Conclusions

This paper presented a novel error estimation method for the milling process based on monitoring data, OMM measurement data, and a neural network. To enhance the model’s performance, we carried out a feature selection process. The top four features identified in Pearson’s correlation coefficient analysis and ten additional features identified in the feature importance plot were extracted from the monitoring data. The MLP model, offering a structure capable of modeling complex nonlinear relationships, was adopted in this study. Cutting force increases due to tool wear progress, which may lead to a discrepancy between the weights of the previously trained model and the data affected by tool wear. We introduced the sliding window approach to consider this phenomenon. The results confirmed that the model denoted a relatively small $R^{2}$ score at the initial stages of training due to insufficient data. However, the $R^{2}$ score increased as time passed, since the model was iteratively trained on complex patterns. We estimated the margin of shape error and its magnitude through the constructed model. Shape error compensation utilizing the tool diameter compensation function provided by a FANUC controller was performed only when the shape error exceeded the tolerance. To define the optimal compensation weight and sliding window size, we compared the results from various conditions. The results showed that optimal performance was achieved with a window size of 4 and a compensation weight of 50%. It was observed that errors occurring during machining did not exceed the tolerance except for the initial section. Hence, it can be concluded that the proposed methodology effectively compensates for shape errors in real time only utilizing monitoring signals.

## Figures and Tables

**Table 1:** List of monitoring data.

Source	Signal Type	Unit
CNC	Spindle speed	min^−1^
	Spindle load	%
	*X*/*Y*/*Z* axis position	mm
	*X*/*Y*/*Z* axis load	%
	*X*/*Y*/*Z* current	mv/A
	Actual feed rate	mm/min
Sensors (DAQ)	Spindle axis current	mA
	Spindle axis voltage	mV
	*X*/*Y*/*Z* axis current	mA
	*X*/*Y*/*Z* axis voltage	mV
	Sound	Pa
	*X*/*Y* axis acceleration	m/s^2^

**Table 2:** Experimental conditions.

Tool Properties	
Type	Flat endmill
Diameter	16 mm
Material	Tungsten carbide (WC-Co)
Number of flutes	2
Workpiece properties	
Material	C45E4
Machining length	120 mm
Machining conditions	
Spindle speed	4100 min^−1^
Feed per tooth	0.1 mm/tooth
Axial depth of cut	2 mm
Radial depth of cut	2 mm

**Table 3:** Hyperparameters setting in MLP Regressor.

Hyperparameter	Optimal Value
Hidden layer1 node	32
Hidden layer2 node	8
Dropout	0.8
Activation function	ReLU
Kernel initializer	He uniform
Optimizer	Adam
Learning rate	0.91
Epochs	1000

## Data Availability

The data presented in this study are available in this article.
